# Diagnostic performance of ^18^F-FDG PET/CT in the prediction of axillary lymph node metastasis in Luminal A breast cancer

**DOI:** 10.1007/s00259-026-07986-0

**Published:** 2026-06-16

**Authors:** Kerim Şeker, Hüsnü Çağrı Genç, Tülay Koç, Mukaddes Yılmaz, Zekiye Hasbek

**Affiliations:** 1 Department of Nuclear Medicine, Faculty of Medicine, Sivas Cumhuriyet University, Sivas, Türkiye; 2 Department of General Surgery, Faculty of Medicine, Sivas Cumhuriyet University, Sivas, Türkiye; 3 Department of Medical Pathology, Faculty of Medicine, Sivas Cumhuriyet University, Sivas, Türkiye; 4 Department of Medical Oncology, Faculty of Medicine, Sivas Cumhuriyet University, Sivas, Türkiye

**Keywords:** Luminal A breast cancer, ^18^F-FDG PET/CT, Axillary lymph node metastasis, Decision curve analysis, Net reclassification improvement (NRI), Integrated discrimination improvement (IDI)

## Abstract

**Purpose:**

This study aims to assess the diagnostic performance of ^18^F-FDG PET/CT parameters for the preoperative prediction of axillary lymph node (ALN) metastasis in Luminal A-type breast cancer patients, and to investigate the net clinical benefit of combining visual analysis with quantitative metrics.

**Materials and methods:**

We retrospectively evaluated 279 treatment-naive female patients histopathologically diagnosed with Luminal A type breast cancer who underwent ^18^F-FDG PET/CT for staging and subsequent ALN sampling between January 2012 and December 2025. Both visual assessment and quantitative parameters—including maximum standardized uptake value (SUVmax), mean standardized uptake value (SUVmean), metabolic tumor volume (MTV), and total lesion glycolysis (TLG) of the primary tumor, as well as axillary SUVmax and the axilla-to-primary SUVmax ratio (SUVr)—were analyzed by two nuclear medicine specialists in consensus. Diagnostic performance was measured using ROC analysis and DeLong’s test. The incremental value of the models was evaluated through Net Reclassification Improvement (NRI) and Integrated Discrimination Improvement (IDI), while clinical utility was determined via Decision Curve Analysis (DCA).

**Results:**

ALN metastasis was confirmed in 152 (54.5%) patients. All quantitative parameters were significantly higher in the metastatic group (p < 0.001). While expert-based visual analysis showed 84.6% accuracy, axillary SUVmax (AUC: 0.881, 95% CI: 0.841–0.920) significantly outperformed visual assessment (AUC: 0.851, p = 0.031). At a cutoff of 0.93, axillary SUVmax yielded 83.6% sensitivity and 79.5% specificity. The Combined Model, integrating visual and quantitative data, achieved the highest diagnostic power (AUC: 0.889) and was statistically superior to axillary SUVmax alone (p = 0.022). The incorporation of quantitative data correctly reclassified 29% (NRI) of metastatic patients initially reported as false negatives in visual analysis and improved overall discrimination by 41.4% (IDI). DCA revealed that the combined model offered a higher net clinical benefit than standard surgical approaches within a 20–90% risk threshold range.

**Conclusion:**

In Luminal A-type breast cancer, objective quantitative 18F-FDG PET/CT parameters and their derived multiparametric models significantly exceed the diagnostic limitations of conventional visual assessment. By providing high net clinical benefit in the surgical decision-making process, the proposed combined model has the potential to serve as a valuable personalized decision support tool for the management of high-risk patients and the de-escalation of invasive surgery in low-risk cohorts.

## Introduction

Breast cancer constitutes the most common invasive malignancy in the female population globally and is among the principal causes of cancer-related mortality [1]. Luminal A-type breast cancer, defined by hormone receptor positivity, HER2 negativity, and low proliferative activity (low Ki-67 index), is the most prevalent subtype and typically exhibits a more indolent clinical course compared to Luminal B and other molecular subtypes [2, 3]. Nevertheless, axillary lymph node involvement continues to be a pivotal prognostic factor, significantly influencing both therapeutic strategies and patient survival [4, 5].

Traditionally performed axillary lymph node dissection (ALND) has been largely replaced by sentinel lymph node biopsy (SLNB) [6, 7]. However, current literature is increasingly debating the potential omission of sentinel lymph node biopsy (SLNB) even in selected early-stage patients with clinically and radiologically negative axilla (cN0), as part of surgical de-escalation strategies [8, 9]. To safely implement this strategy, the absence of axillary metastasis must be demonstrated with high accuracy using non-invasive methods [10].

Axillary ultrasonography (USG) and mammography are the standard imaging modalities for detecting lymph node metastases [4]. However, ^18^F-Fluorodeoxyglucose (^18^F-FDG) positron emission tomography/computed tomography (PET/CT) can contribute to staging—particularly in suspicious cases—by providing metabolic information about the tumor in addition to anatomical data [11, 12]. While the role of ^18^F-FDG PET/CT in aggressive breast cancer subtypes (Triple Negative and HER2+) is well-established, literature regarding its diagnostic performance and the efficacy of its parameters in Luminal A-type tumors—which exhibit lower metabolic activity—remains sparse and inconsistent [13, 14].

In ^18^F-FDG PET/CT imaging, the glucose avidity of tumor cells is evaluated in addition to morphological findings. Luminal A-type tumors exhibit lower FDG uptake compared to more aggressive subtypes; while this may lead to decreased sensitivity, it typically ensures high specificity. In this patient cohort, employing detailed quantitative analyses rather than relying solely on visual assessment may contribute to a more accurate evaluation of the extent of disease spread [12, 15, 16].

Furthermore, semi-quantitative metrics derived from the primary tumor on ^18^F-FDG PET/CT images facilitate the quantification of imaging findings. Parameters such as maximum standardized uptake value (SUVmax), mean standardized uptake value (SUVmean), metabolic tumor volume (MTV), and total lesion glycolysis (TLG) are semi-quantitative values calculable from 18F-FDG PET/CT scans, which significantly contribute to the assessment of tumor burden and aggressiveness. Previous studies across various malignancies have established that primary tumors characterized by elevated SUVmax, MTV, and TLG values are associated with a higher risk of metastasis [17–19].

While the utility of ^18^F-FDG PET/CT in aggressive breast cancer subtypes has been extensively investigated, research specifically focusing on the predictive value of volumetric parameters—such as SUVmax, MTV, and TLG—for axillary metastasis in Luminal A patients remains limited, despite this being the most prevalent yet metabolically challenging subtype. The aim of this study is to evaluate the correlation between preoperative ^18^F-FDG PET/CT metabolic parameters and pathological axillary nodal status in patients with Luminal A breast cancer. We anticipate that our findings will provide objective criteria to assist clinicians in predicting axillary metastasis non-invasively, thereby facilitating informed decisions on surgical de-escalation and addressing a significant gap in the current literature.

## Materials and methods

This study was conducted using a retrospective design. The study protocol was approved by the local ethics committee (Approval No: 2026-02/45), and all procedures were performed in accordance with the principles of the Declaration of Helsinki.

### Patient selection criteria

Medical records of patients diagnosed with breast cancer between January 1, 2012, and December 31, 2025, were retrospectively screened using the hospital information system. Adult patients (aged ≥ 18 years) who were histopathologically diagnosed with Luminal A-type breast cancer—defined as estrogen receptor and progesterone receptor-positive, HER2-negative, and a Ki-67 index of < 14—were included in the study. All included patients had undergone ^18^F-FDG PET/CT for initial staging followed by axillary lymph node sampling and were treatment-naive, having received no prior surgery, neoadjuvant or adjuvant chemotherapy, or radiotherapy before imaging.

Patients were excluded if they had molecular subtypes other than Luminal A, lacked a staging ^18^F-FDG PET/CT scan, had undergone surgical intervention or systemic therapy prior to the PET/CT scan, or presented with a secondary malignancy,

### Histopathological evaluation

The Luminal A subtype was confirmed by extracting the age at diagnosis, hormone receptor status (ER, PR), Ki-67 proliferation index, and HER2 expression levels from the patients’ pathology reports. ALN sampling was performed utilizing either SLNB or ALND, depending on the clinical and radiological axillary status of the patients. For SLNB, lymphatic mapping was performed using solely the blue dye method. The harvested lymph nodes were bisected and evaluated using standard hematoxylin and eosin (H&E) staining. In cases where standard H&E evaluation was equivocal, immunohistochemical staining (e.g., for cytokeratin) was applied to detect isolated tumor cells or micrometastases. Based on the final histopathological examination results, the axillary status was categorically dichotomized into two groups: reactive (absence of metastasis) and metastatic (presence of macrometastasis or micrometastasis).

### ^18^F-FDG PET/CT imaging protocol

All patients underwent a minimum of 6 h of fasting, ensuring blood glucose levels were below 180 mg/dL prior to the procedure. ^18^F-FDG was administered intravenously at a dose of 0.1 mCi/kg, and imaging was initiated approximately 60 min post-injection. All scans were performed using a Discovery 600 PET/CT scanner (GE Medical Systems, USA). For attenuation correction, a non-contrast CT scan was performed using 70 mA, 120 kV, and a 2.5 mm slice thickness. PET images were acquired in 3D mode from the vertex to the mid-thigh, with an acquisition time of 2 min per bed position. The system’s intrinsic spatial resolution (Full Width at Half Maximum [FWHM]) was approximately 5 mm, and the post-reconstruction voxel size was 1.3 mm.

The ^18^F-FDG PET/CT images were evaluated both visually and quantitatively by two experienced nuclear medicine specialists. For quantitative assessment, separate volumes of interest (VOIs) were placed to encompass the primary tumor and the axillary lymph nodes. An adaptive threshold of 42% of the maximum lesional metabolic activity was utilized for the PET images. The SUVmax, SUVmean, and MTV values of the primary tumor were automatically calculated using the specialized software (PET REV) on the workstation. TLG was derived by multiplying the MTV by the SUVmean.

In addition to primary tumor metrics, the SUVmax of the axillary lymph nodes was measured. The axilla-to-primary SUVmax ratio (SUVr) was subsequently calculated by dividing the axillary lymph node SUVmax by the primary tumor SUVmax. Furthermore, axillary lymph nodes were visually assessed through a joint consensus between the two nuclear medicine physicians and categorized as either reactive or metastatic.

### Statistical analysis

Statistical analyses were performed using IBM SPSS Statistics (Version 26.0) and R software (Version 4.5.2; employing pROC, nricens, and dcurves packages). The normality of variables was assessed using the Shapiro-Wilk and Kolmogorov-Smirnov tests. Since the data were not normally distributed, continuous variables were presented as medians and interquartile ranges (IQR), while categorical variables were reported as frequencies and percentages. The Mann-Whitney U test was utilized to compare quantitative ^18^F-FDG PET/CT parameters between the metastatic and reactive groups. The diagnostic performance of the visual assessment conducted by nuclear medicine specialists was determined through sensitivity, specificity, and overall accuracy rates. The diagnostic agreement between the experts’ consensus visual analysis and the gold-standard histopathological results was evaluated using Cohen’s Kappa (κ) test. The predictive success of ^18^F-FDG PET parameters for axillary metastasis was assessed via Receiver Operating Characteristic (ROC) curve analysis, and optimal threshold values were identified using the Youden index. DeLong’s test was employed to compare the diagnostic accuracy of the axillary SUVmax parameter with that of visual analysis. The incremental value of adding quantitative data to visual analysis for diagnostic classification was measured using Net Reclassification Improvement (NRI) and Integrated Discrimination Improvement (IDI). Specifically, NRI was utilized to quantify the proportion of patients correctly reassigned to a more accurate diagnostic category (e.g., metastatic patients correctly reclassified from false-negative to true-positive) by the addition of quantitative metrics. IDI was used to evaluate the overall enhancement in the model’s predicted probabilities, reflecting the continuous improvement in discrimination between metastatic and reactive cases. To identify independent risk factors, parameters found to be significant in univariate analysis were included in a multivariate logistic regression model, and a “Combined Model” was developed based on the resulting probability coefficients. Finally, the net benefit provided by the developed models to clinical decision-making processes was evaluated through Decision Curve Analysis (DCA). For all analyses, a p-value of < 0.05 was considered statistically significant.

## Results

### Patient demographics and histopathological findings

A total of 279 female patients were included in the study. The mean age of the patients was 54.5 ± 12.3 years (range: 28–87). Histopathological evaluation revealed axillary lymph node metastasis in 152 (54.5%) patients, while reactive changes were observed in 127 (45.5%) patients.

### Comparison ^18^F-FDG PET/CT parameters

In the comparison between metastatic and reactive groups, all primary tumor metabolic (SUVmax, SUVmean) and volumetric (MTV, TLG) parameters, as well as axillary metrics (axillary SUVmax and SUVr), were found to be statistically significantly higher in the metastatic group (*p* < 0.001). Detailed quantitative data for both groups are presented in Table 1. This fundamental comparison initially confirms that quantitative ^18^F-FDG PET/CT data serve as strong potential biomarkers for differentiating axillary nodal status.

**Table 1:** Comparison of ^18^F-FDG PET/CT parameters between metastatic and reactive axillary lymph node groups

Parameters	Reactive group (*n* = 127) [Median (IQR)]	Metastatic group (*n* = 152) [Median (IQR)]	p*
Primary SUVmax	3.55 (2.65)	4.79 (4.33)	0.001
Primary SUVmean	2.07 (1.78)	2.91 (2.81)	0.001
Primary MTV (cm3)	3.94 (4.93)	6.04 (8.35)	< 0.001
Primary TLG (g)	8.61 (16.09)	18.46 (27.64)	< 0.001
Axillary SUVmax	0.72 (0.30)	2.20 (3.22)	< 0.001
SUVr	0.20 (0.14)	0.52 (0.57)	< 0.001

Note: Data are presented as Median (Interquartile Range - IQR)

*p-values were calculated using the Mann-Whitney U test; *p* < 0.05 was considered statistically significant

*SUV* Standardized Uptake Value, *MTV *Metabolic Tumor Volume, *TLG *Total Lesion Glycolysis, *SUVr* Axilla-to-primary standardized uptake value ratio

### Visual assessment

In the visual analysis performed by the consensus of two nuclear medicine specialists, 133 (47.7%) out of the 279 patients were classified as positive (suggestive of metastasis), while 146 (52.3%) were classified as negative (suggestive of reactive changes). The evaluation based on expert opinion demonstrated a sensitivity of 79.6%, a specificity of 90.6%, and an overall diagnostic accuracy of 84.6% (Table 2). A statistically significant and substantial level of agreement was observed between the experts’ visual assessments and the histopathological results (κ = 0.693, *p* < 0.001).

**Table 2:** Comparative diagnostic performance of visual analysis and quantitative ^18^F-FDG PET/CT parameters in predicting axillary metastasis in Luminal-A breast cancer

Parameters	AUC	95% CI	*p*	Cut-off	Sensitivity (%)	Specificity (%)
Combined Model	0.889	0.852–0.926	< 0.001	*	*	*
Axillary SUVmax	0.881	0.841–0.920	< 0.001	0.93	83.6	79.5
Visual Assessment	0.851	0.810–0.892	< 0.001	-	79.6	90.6
SUVr	0.825	0.777–0.874	< 0.001	0.36	65.8	89
Primary TLG	0.665	0.602–0.729	< 0.001	8.62	76.3	51.2
Primary MTV	0.633	0.568–0.698	< 0.001	5.86	54.6	69.3
Primary SUVmean	0.616	0.550–0.682	0.001	2.8	52.6	66.9
Primary SUVmax	0.613	0.547–0.679	0.001	4.66	52	68.5

The diagnostic performance data presented in the table encompass the Area Under the Curve (AUC), Maximum Standardized Uptake Value (SUVmax), Mean Standardized Uptake Value (SUVmean), Metabolic Tumor Volume (MTV), Total Lesion Glycolysis (TLG), Axilla-to-primary SUVmax ratio (SUVr), Confidence Intervals (CI), Sensitivity and Specificity values, and Receiver Operating Characteristic (ROC) curve parameters

Statistical analyses, validated by the DeLong test (*p* = 0.031), demonstrated that the diagnostic success of axillary SUVmax was significantly superior to visual assessment

Furthermore, the combined model, which integrates visual and quantitative data, further enhanced diagnostic accuracy compared to the use of SUVmax alone (*p* = 0.022)

Additionally, Net Reclassification Improvement (NRI) data documented that 29% of metastatic patients who were not detected by visual analysis could be correctly reclassified using the established axillary SUVmax threshold of 0.93

Note on the Combined Model: Since the combined model is a probability estimation model developed through logistic regression analysis of visual assessment and axillary SUVmax data, a single physical cutoff value, sensitivity, or specificity has not been specified for this parameter

### Diagnostic performance in predicting axillary metastasis

**Fig. 1:**
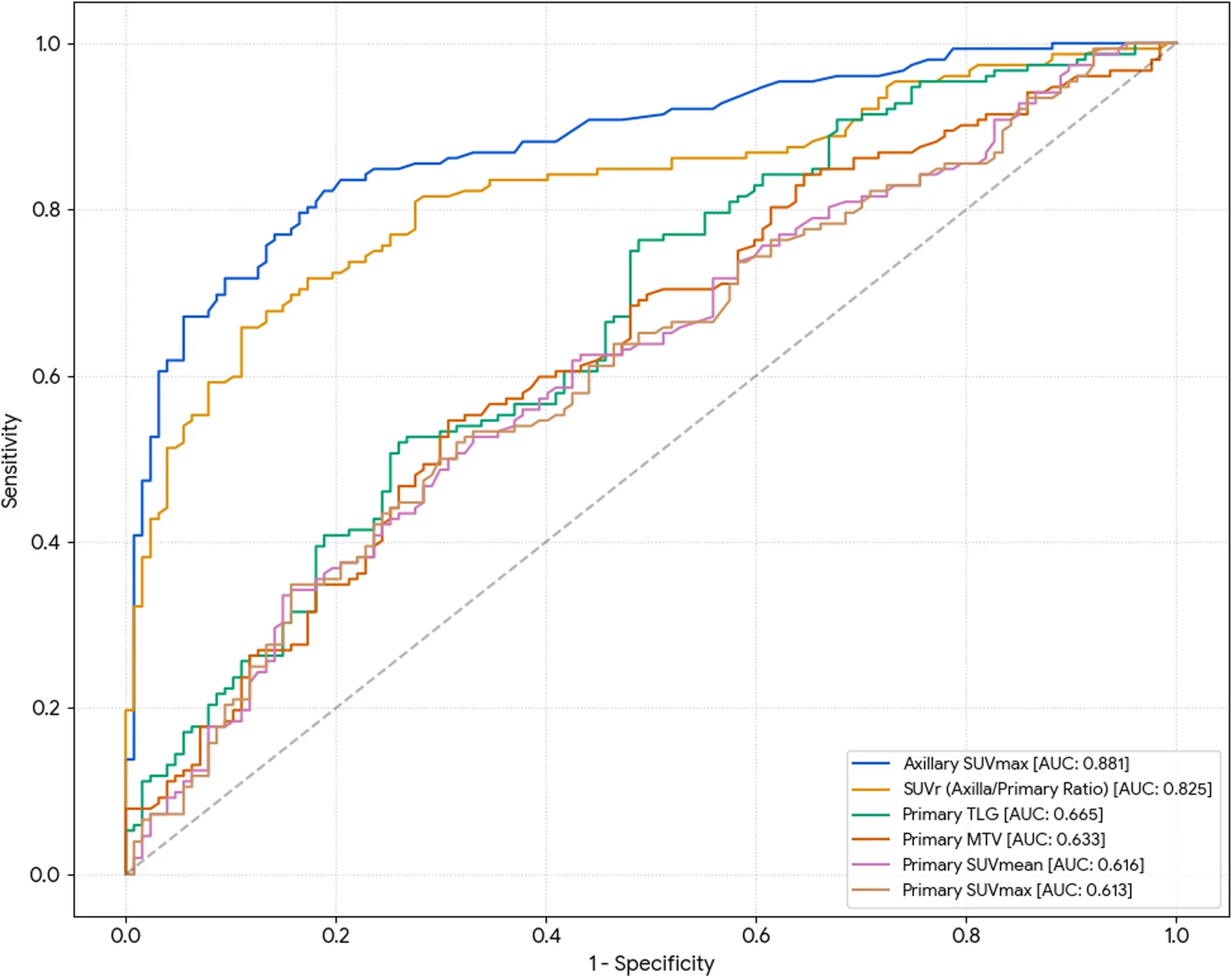
Comparative ROC curve analysis of quantitative ^18^F-FDG PET/CT parameters. Receiver Operating Characteristic (ROC) curve analysis demonstrating the diagnostic performance of various quantitative ^18^F-FDG PET/CT parameters in predicting axillary lymph node metastasis in patients with Luminal-A breast cancer. Among the evaluated metrics, Axillary SUVmax and SUVr (Axilla-to-Primary Ratio) achieved the highest Area Under the Curve (AUC) values, exhibiting the most robust diagnostic accuracy for detecting metastasis. It is observed that the predictive performance of primary tumor-related metabolic (SUVmax, SUVmean) and volumetric (MTV, TLG) parameters is lower compared to the quantitative data derived directly from the axillary region. Abbreviations: ROC, receiver operating characteristic; AUC, area under the curve; SUVmax, maximum standardized uptake value; SUVmean, mean standardized uptake value; MTV, metabolic tumor volume; TLG, total lesion glycolysis; SUVr, axilla-to-primary SUVmax ratio

All evaluated ^18^F-FDG PET/CT parameters were found to be statistically significant predictors of axillary lymph node metastasis (*p* < 0.001). Among these, axillary SUVmax exhibited the highest diagnostic performance, with an AUC of 0.881 (95% CI: 0.841–0.920). At an optimal cutoff value of 0.93, axillary SUVmax yielded a sensitivity of 83.6% and a specificity of 79.5%. SUVr was the parameter that reached the highest specificity (89.0%) at a threshold of 0.36 (AUC: 0.825). Conversely, the predictive success of primary tumor metabolic and volumetric parameters (TLG, MTV, SUVmax, and SUVmean) was notably lower (AUC range: 0.613–0.665) compared to axillary-focused metrics. The comparative diagnostic performances of these parameters are detailed in Table 2 and illustrated in Fig. 1.

### Clinical improvement and discriminative power analysis of quantitative parameters

**Fig. 2:**
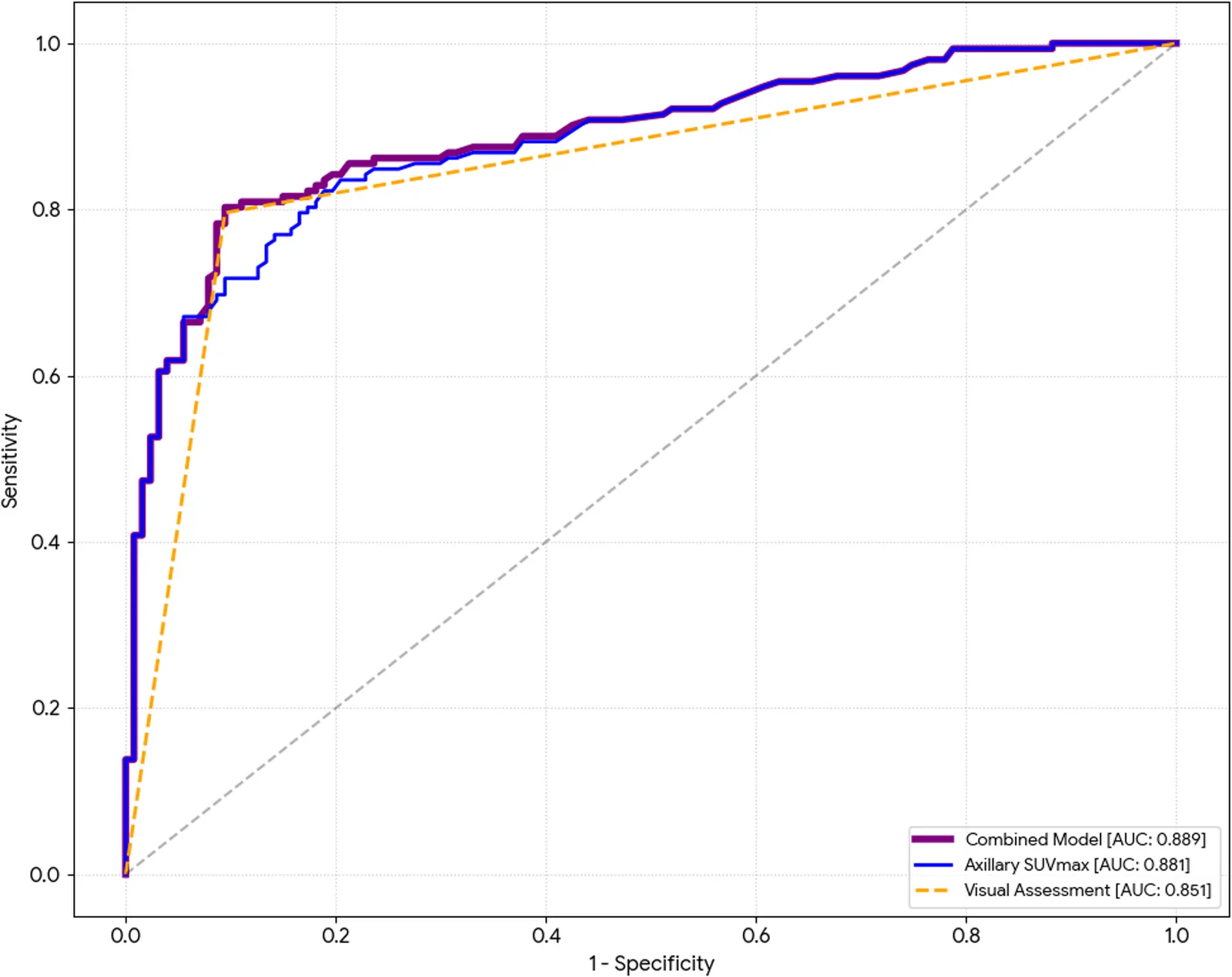
Comparative ROC curve analysis of diagnostic models for predicting axillary metastasis. Comparison of the diagnostic performances of visual assessment, quantitative axillary SUVmax, and the combined model—developed by integrating these two parameters via logistic regression analysis—in detecting axillary lymph node metastasis in patients with Luminal A breast cancer. The combined model, incorporating both visual and quantitative data (solid purple line), achieved the highest accuracy with an AUC of 0.889. Quantitative axillary SUVmax alone (AUC: 0.881) significantly outperformed the visual assessment performed by the nuclear medicine specialist (AUC: 0.851) (DeLong test, p = 0.031). The integration of both modalities (combined model) significantly enhanced the diagnostic power compared to the use of quantitative data alone (DeLong test, p = 0.022). The central diagonal dashed line represents the reference line of no diagnostic value. Abbreviations: ROC, receiver operating characteristic; AUC, area under the curve; SUVmax, maximum standardized uptake value. Note: The ‘Combined Model’ represents the logistic regression probability score integrating the consensus visual assessment with the quantitative axillary SUVmax

According to the DeLong test results, the diagnostic performance of the axillary SUVmax parameter (AUC: 0.881) was found to be statistically significantly superior to that of visual assessment (AUC: 0.851; *p* = 0.031) (Fig. 2). To evaluate the clinical implications of this quantitative superiority, Net Reclassification Improvement (NRI) analysis was performed. The results revealed that 9 out of 31 metastatic patients (29%) who were incorrectly identified as negative (reactive) in the visual analysis were correctly reclassified using only the SUVmax threshold of 0.93. Furthermore, Integrated Discrimination Improvement (IDI) analysis confirmed that the integration of quantitative data into the model enhanced the discriminative power between metastatic and reactive cases by 41.4%.

### Multivariate logistic regression and clinical net benefit assessment via decision curve analysis

In the multivariate logistic regression model established to predict axillary metastasis, a positive visual assessment was found to increase the risk of metastasis by 12.74 times (OR = 12.746, *p* < 0.001). Independently of this, each unit increase in axillary SUVmax was associated with a 1.73-fold increase in the probability of metastasis (OR = 1.737, *p* = 0.031). The Combined Model, developed by integrating these two parameters, achieved the highest diagnostic performance in the study (AUC: 0.889). Comparative analysis using DeLong’s test revealed that the performance of the combined model was statistically significantly superior even to the SUVmax-only model (AUC: 0.881; *p* = 0.022).

**Fig. 3:**
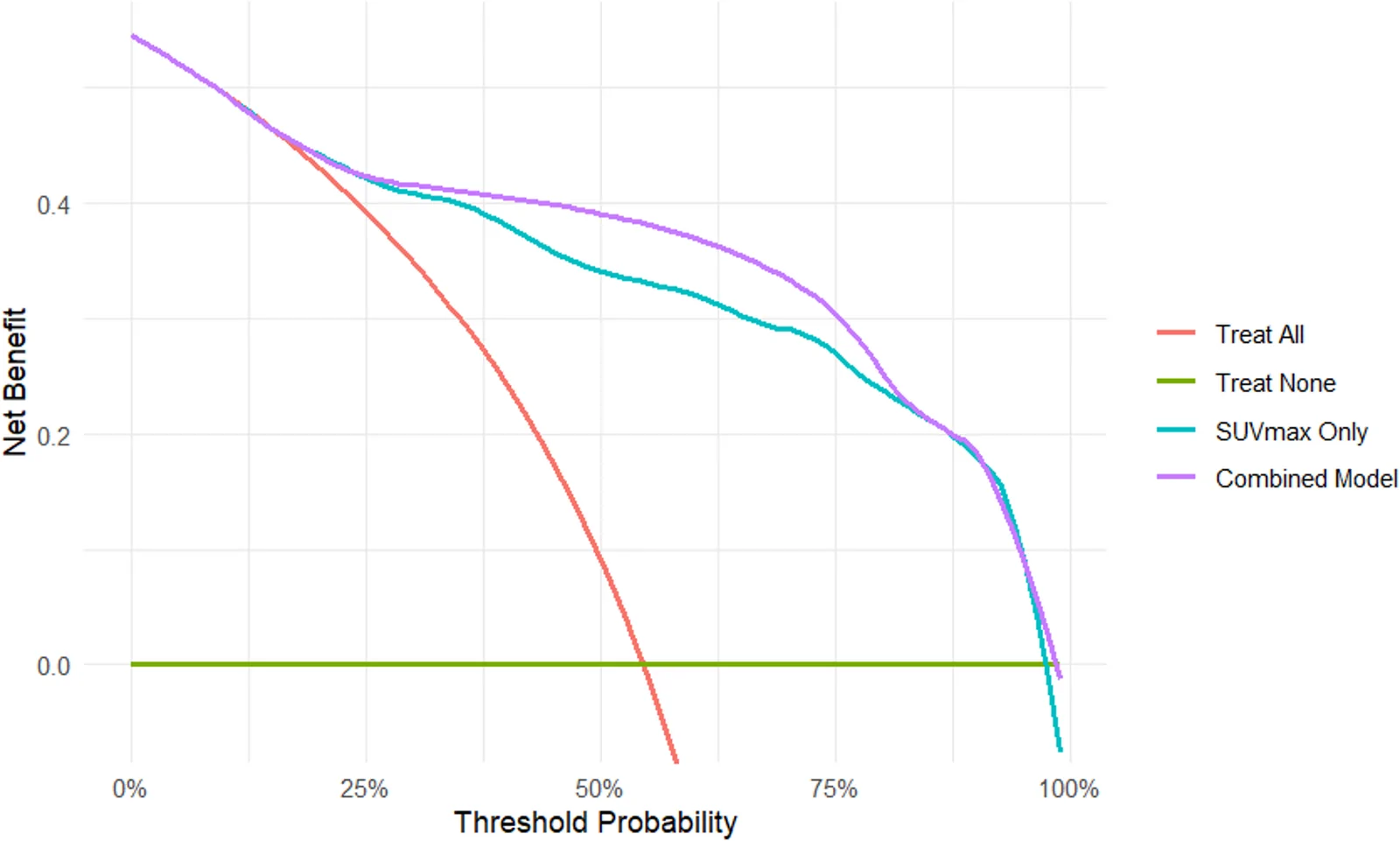
Decision Curve Analysis (DCA) demonstrating the clinical net benefit of models in predicting axillary metastasis. Decision Curve Analysis (DCA) comparing the clinical utility of the axillary SUVmax model and the combined model (visual assessment + axillary SUVmax) for predicting axillary lymph node metastasis in patients with Luminal A breast cancer. The combined model, represented by the solid purple line, exhibits a higher net clinical benefit across a wide threshold probability range of approximately 20% to 90% compared to both the SUVmax model alone and standard clinical strategies (Treat All and Treat None). These results demonstrate that the integration of visual and quantitative data enhances the capacity for accurate staging while minimizing unnecessary invasive procedures (e.g., lymph node dissection) during surgical planning, thereby serving as a robust clinical decision-support tool. Abbreviations: DCA, decision curve analysis; SUVmax, maximum standardized uptake value. In this graph, ‘Treat All’ assumes all patients have metastasis and undergo axillary surgery, while ‘Treat None’ assumes no patients have metastasis. The ‘Net Benefit’ (y-axis) reflects the clinical balance between correctly identifying metastatic nodes and avoiding unnecessary surgeries. The ‘Threshold Probability’ (x-axis) represents the risk level at which a clinician or patient would opt for an invasive staging procedure

Decision Curve Analysis (DCA) further confirmed that the combined model, which integrates visual and quantitative data, provided a higher net clinical benefit than both the standalone SUVmax model and standard surgical approaches across a wide risk threshold range of approximately 20% to 90% (Fig. 3). These findings supports the integrating quantitative ^18^F-FDG PET/CT metrics into clinical decision-making processes optimizes axillary staging accuracy and patient management in Luminal A-type breast cancer.

## Discussion

This study evaluated the diagnostic performance of preoperative ^18^F-FDG PET/CT in patients with Luminal A-type breast cancer and demonstrated that the integration of conventional visual assessment with quantitative metabolic data yields results that are statistically and clinically superior to individual parameters in predicting axillary metastasis.

The findings of this study demonstrate the critical role of metabolic characteristics of both the primary tumor and axillary lymph nodes in predicting metastasis within a homogeneous patient cohort consisting exclusively of the Luminal A subtype—a group known for having the most limited metabolic activity in ^18^F-FDG PET/CT imaging. As summarized in the systematic review by Hwang et al., much of the existing literature comprises heterogeneous series where various molecular subtypes are evaluated together. Such heterogeneity can weaken the statistical power of the relationship between primary tumor metabolic parameters and axillary status. Indeed, the fact that the primary tumor SUVmax attained statistical significance for predicting metastasis in only one-third of the 18 studies reviewed by Hwang et al. reflects the inconsistency and uncertainty created by such cohort heterogeneity [20]. In contrast, despite focusing solely on Luminal A cases characterized by low ^18^F-FDG uptake, our study helps fill this void in the literature by clearly demonstrating a robust and significant association between quantitative parameters—of both the primary tumor and axillary lymph nodes—and metastasis. Unlike mixed series, this refined approach reveals that the diagnostic power of ^18^F-FDG PET/CT can be demonstrated with methodological precision even in the most metabolically silent molecular subtype.

One of the hallmark findings of our study is the highly significant correlation between metastasis and both primary tumor volumetric metrics (MTV, TLG) and axillary quantitative parameters (SUVmax and SUVr) within our Luminal A cohort. This is particularly noteworthy given that Luminal A is the most challenging subtype to evaluate on ^18^F-FDG PET/CT due to its low cell proliferation rate and subsequent limited radiopharmaceutical uptake. Regarding the definition of the Luminal A subtype, we acknowledge that recent iterations of the St. Gallen consensus [21, 22] have proposed a higher Ki-67 threshold (typically < 20%) to accommodate inter-laboratory variability in Ki-67 scoring. We deliberately applied the more restrictive < 14% criterion to maximize the biological homogeneity of our cohort and isolate the metabolic challenges unique to truly indolent Luminal A tumors, thereby minimizing potential confounding by borderline, more metabolically active Luminal B-like cases [22]. Our results are fundamentally consistent with the findings of Sayed et al., who demonstrated that metabolic tumor volume serves as an independent predictor of occult axillary metastasis in early-stage luminal cases [23]. Nevertheless, the present research advances the current literature by establishing that focusing solely on primary tumor features is insufficient; instead, axillary SUVmax and the SUVr play a pivotal role in the predictive hierarchy. By demonstrating that the volumetric load of the primary lesion and the metabolic intensity of the axillary node serve as effective composite biomarkers, our results underscore the importance of a multi-faceted quantitative approach when risk-stratifying nodal spread in metabolically indolent subtypes.

The observation that ^18^F-FDG PET/CT demonstrated limited sensitivity but high specificity during the axillary evaluation—despite the consensus visual interpretation by two experienced nuclear medicine specialists—is in complete alignment with current meta-analysis data. The sensitivity of 52.2% and specificity of 91.6% reported in the systematic review by Kasem et al. reflect that the primary strength of this modality lies in confirming metastasis (ruling in) rather than excluding it (ruling out) [24]. Similarly, a meta-analysis by Zhang et al. revealed that both ^18^F-FDG PET/CT and Magnetic Resonance Imaging (MRI) exhibit a comparable profile of low sensitivity [25]. In routine clinical practice, axillary USG unequivocally remains the primary, most accessible, and cost-effective first-line imaging modality for preoperative nodal staging. Although our study did not directly compare PET/CT with USG, we do not propose ^18^F-FDG PET/CT as a replacement for standard axillary USG. Instead, our proposed combined quantitative model is best utilized as a complementary tool. It offers significant clinical value particularly in cases where USG and mammography findings are equivocal or indeterminate, providing an objective, multiparametric metabolic evaluation to help guide complex surgical de-escalation decisions. Furthermore, the study by Güneş et al. confirms that the Luminal A subtype is the most misleading group for nuclear medicine specialists, yielding the lowest sensitivity levels due to its inherently low proliferation index [26].

It is precisely at this point that the most unique contribution of our study emerges. While the majority of the literature reports overall success in mixed patient populations, we specifically focused on the Luminal A subgroup—the weakest link for ^18^F-FDG PET/CT—aiming to overcome existing diagnostic barriers through quantitative data. We successfully surpassed the diagnostic ceiling of expert-based visual assessment by incorporating objective parameters, such as axillary SUVmax and, more importantly, the axilla-to-primary SUVmax ratio (SUVr). The relatively high diagnostic accuracy achieved in our study, despite the inherently low ^18^F-FDG avidity of Luminal A tumors, can be directly attributed to this methodological shift. While low-intensity metastatic uptake in axillary nodes often blends into background tissue and eludes conventional visual detection, utilizing derived quantitative thresholds (such as an axillary SUVmax of 0.93) amplifies these subtle metabolic differences. Furthermore, the use of SUVr normalizes the nodal activity against the primary tumor’s baseline low activity, effectively unmasking occult metastases by using the tumor’s own biology as an internal reference. The traditional binary reporting system (positive/negative) prevalent in the literature often leads to the oversight of low-activity metastases typical of Luminal A tumors. The axillary SUVmax thresholds identified in our study provide a quantitative standardization for low-intensity metabolic activity that may elude the human eye, effectively transforming subjective experience into objective evidence. The optimal axillary SUVmax cutoff of 0.93 identified in our study may appear numerically low compared to more aggressive breast cancer subtypes; however, this accurately reflects the low cellular proliferation rate (Ki-67 < 14% in our cohort) and limited ^18^F-FDG avidity inherently characteristic of the Luminal A subtype. Notably, the fact that SUVr reached the highest specificity by offering a comparison independent of the primary tumor’s biological behavior confirms its potential as one of the most reliable biomarkers for minimizing unnecessary invasive procedures in Luminal A patients.

The determination of optimal cutoff values for axillary SUVmax via ROC curve analysis and the direct comparison of this performance with visual assessment are in full methodological alignment with the existing literature seeking standardized thresholds for semi-quantitative ^18^F-FDG PET/CT parameters. Furthermore, the review by Hwang et al. highlights that the SUVmax of the axillary lymph node is one of the most robust indicators for determining metastatic status [20].

The most concrete achievement of our study in this field is that the axillary SUVmax value yielded a higher Area Under the Curve (AUC: 0.881) compared to visual interpretation, a difference that was statistically validated by the DeLong test (*p* = 0.031). This finding is consistent with the results obtained by researchers such as Li and Cheng in their multiparametric models. Notably, Cheng et al. achieved high AUC values ranging from 0.93 to 0.94 by integrating specialized high-resolution PET/CT imaging and clinical features into machine learning models [27]. Similarly, Li et al. moved beyond the limitations of single parameters by developing a comprehensive model that integrated clinicopathological data, ultrasound findings, and ^18^F-FDG PET/CT radiomics scores, thereby elevating diagnostic accuracy to an AUC of 0.87 [28].

The data presented in our study demonstrate that routine semi-quantitative metrics—specifically optimized thresholds such as 0.93—are capable of surpassing the diagnostic boundaries of expert-based visual interpretation without necessitating sophisticated radiomics analyses or complex machine learning algorithms. This provides scientific validation that subjecting axillary ^18^F-FDG PET/CT findings to objective quantitative standardization, rather than a purely subjective present versus absent evaluation, significantly enhances diagnostic reliability, particularly in demanding scenarios such as Luminal A-type breast cancer.

One of the most significant methodological contributions of our study to the literature is that we did not merely report ^18^F-FDG PET/CT data as static diagnostic values. Instead, we quantified the tangible impact of integrating these quantitative parameters into visual assessment using Net Reclassification Improvement (NRI) and Integrated Discrimination Improvement (IDI) analyses. While existing literature acknowledges that multiparametric models generally outperform individual imaging findings, the exact clinical implications of this superiority—specifically what it corresponds to in daily practice—are rarely reported [27–30].

Our results demonstrate that incorporating routine semi-quantitative data, such as axillary SUVmax, into the diagnostic model provides a substantial gain in classification accuracy, obviating the need for advanced radiomic features. Specifically, the 29% improvement rate revealed by our NRI analysis indicates that approximately one-third of metastatic cases visually classified as reactive were accurately identified solely through the application of quantitative thresholds. Furthermore, the 41.4% increase in IDI confirms the dramatic extent to which quantitative data enhances the discriminative sharpness between metastatic and reactive cases.

The most critical output of our study regarding clinical application is solidified by DCA. The concept of net clinical benefit, emphasized by Li et al. in their radiomics-based models [28], was demonstrated in our study in favor of the Combined Model (integrating visual and quantitative assessments) across a wide risk threshold range of approximately 20% to 90%. This wide threshold range was intentionally evaluated to reflect the diverse risk tolerances encountered in clinical practice. The decision to proceed with invasive axillary staging varies significantly; some clinicians and patients may opt for surgery even at a lower risk of metastasis (e.g., 20%), whereas others might require a much higher probability (e.g., 90%) to justify the potential morbidity of the procedure. This finding reveals that combining the expertise of the nuclear medicine physician with objective quantitative data provides a far more reliable guide in the surgical decision-making process than the standard Treat All (operating on everyone) or Treat None (operating on no one) strategies. The net benefit observed—carrying the potential to reduce unnecessary sentinel lymph node biopsies (SLNB) or high-morbidity axillary dissections in early-stage Luminal-A patients— suggests the potential utility of quantitative models in personalized surgical management.

### Study limitations

Despite the robust clinical data and advanced statistical analyses presented in our study, several limitations must be considered when interpreting the findings. First, the retrospective nature of the research carries an inherent risk of selection bias during the data collection process. However, as a rare occurrence in the literature, this design uniquely enabled the meticulous and longitudinal examination of a highly homogeneous and extensive cohort focused exclusively on the Luminal A subtype.

Secondly, the single-center design of our study and the lack of an external validation cohort may limit the external validity and generalizability of the established cutoff values and diagnostic models. Future multicenter prospective studies are required to externally validate the proposed threshold values. Conversely, this structure facilitated a high degree of standardization across all ^18^F-FDG PET/CT protocols—including post-injection uptake times and equipment calibration—as well as surgical techniques and histopathological evaluation processes. By eliminating potential heterogeneity arising from inter-institutional practice variations, this approach significantly enhanced the internal consistency of our findings.

Finally, the moderate performance of even the most potent quantitative predictors of axillary metastasis is intrinsically linked to the spatial resolution limits of current PET systems. The approximately 5 mm resolution threshold of contemporary PET technology renders the physical detection of micrometastases (0.2–2.0 mm) particularly challenging, especially in tumors with low metabolic activity such as the Luminal A subtype. This observation is consistent with literature indicating that no single ^18^F-FDG PET/CT parameter can currently serve as a definitive rule-out tool, and the modality has not yet reached the level of a fully reliable triage test for nodal staging.

The findings of our study offer a nuanced contribution to this landscape, specifically within the context of Luminal A-type breast cancer. While the application of axillary thresholds and our proposed combined model demonstrates the potential to minimize unnecessary invasive procedures, the question of whether negative ^18^F-FDG PET/CT findings can safely justify forgoing axillary surgery—specifically SLNB—remains a subject of ongoing debate, fundamentally constrained by inherent biological resolution limits.

## Conclusion

This study provides robust evidence that shifts the axillary staging process from subjective visual interpretation toward objective quantitative standardization in Luminal A-type breast cancer—a subgroup historically characterized by the lowest sensitivity in nuclear medicine imaging. Our findings demonstrate that a Combined Model, which integrates expert experience with objective parameters such as axillary SUVmax and the axilla-to-primary ratio (SUVr), significantly enhances diagnostic precision and yields the highest clinical net benefit in surgical decision-making, as substantiated by Decision Curve Analysis (DCA).

While the inherent spatial resolution limits of current PET/CT technology prevent this methodology from serving as a definitive rule-out tool to entirely replace invasive surgery, this multiparametric approach can serve as a valuable personalized decision-support mechanism. It has the potential to assist in surgical planning for high-risk patients and provides a rigorous scientific foundation for surgical de-escalation strategies in low-risk populations.

## Data Availability

The datasets generated during and/or analysed during the current study are available from the corresponding author on reasonable request.
